# Editorial Comment: Secondary polycythemia in men receiving testosterone therapy increases risk of major adverse cardiovascular events and venous thromboembolism in the first year of therapy

**DOI:** 10.1590/S1677-5538.IBJU.2023.01.04

**Published:** 2023-01-26

**Authors:** Rodrigo de Castro Barros

**Affiliations:** 1 Universidade Federal Fluminense Hospital Universitário Antônio Pedro Serviço de Urologia Niterói RJ Brasil Serviço de Urologia, Hospital Universitário Antônio Pedro - Universidade Federal Fluminense - UFF, Niterói, RJ, Brasil

Jesse Ory^1, 2^, Sirpi Nackeeran^3^, Navin C. Balaji^3^, Joshua M. Hare^4^, Ranjith Ramasamy^1^

**J Urol. 2022 Jun;207(6):1295-1301.**

**DOI: 10.1097/JU.0000000000002437 | ACCESS: 35050717**

## COMMENT

The evidence is inconsistent about the association between testosterone therapy (TT) and subsequent risk of cardiovascular events (1). According to some guidelines, such as those of the American Urological Association (AUA), we should measure hemoglobin and hematocrit and inform patients about the increased risk of polycythemia before offering TT (2). According to the European Urological Association (EAU) guidelines, a hematocrit (HCT) > 54% should require testosterone therapy withdrawal, reduction of dose, change of formulation or venesection to avoid any cardiovascular events (3). However, it is not known if TT increases the risk of cardiovascular and thromboembolic events and what the safe hematocrit cutoff value is.

In this study, the authors tried to find the unsafe hematocrit threshold for men receiving TT and determine whether secondary polycythemia among men receiving TT causes an increased risk of major adverse cardiovascular events (MACE) and venous thromboembolic events (VTE). They performed a retrospective cohort study from a database of 74 million people including two groups of men with low testosterone who received TT and subsequently either did or did not develop polycythemia, and compared 5,842 men in each group. Polycythemia was defined as a hematocrit above 52%, in keeping with the AUA guideline definition. The primary outcome was incidence of MACE and VTE in the first year of TT. The authors found that men on TT who developed secondary polycythemia had a higher incidence risk of MACE and VET than men who did not develop polycythemia. Moreover, they reported that hypogonadal men on TT versus those off testosterone had similar rates of MACE/VTE in the absence of polycythemia (4).

This study has some limitations. The TT group consisted of men who received two prescriptions for TT within nine months and the authors did not specify what modalities of testosterone were used (ex. gel, shorter- or longer-acting injection). Baseline hematocrit was greater in the polycythemia group (47.4%) versus the non-polycythemia group (42.5%). Nevertheless, I congratulate everyone involved in this study, which is the first to establish secondary polycythemia from TT as an independent risk factor for MACE/VTE using a specific hematocrit-based cutoff. This cutoff can guide our clinical practice and we can tell patients undergoing TT that they are at a higher risk of MACE and VTE if their hematocrit reaches or exceeds 52% during the first year of therapy. Despite these findings, further studies are needed to confirm the association between TT dosage and patient adherence to secondary polycythemia and MACE/VTE.
